# The Technique of Prostatectomy*Read before the Chattahoochee Valley Medical Association, Jan. 16-17, 1912.

**Published:** 1912-01

**Authors:** Edgar G. Ballenger, Omar F. Elder

**Affiliations:** Atlanta, Ga.; Atlanta, Ga.


### THE TECHNIQUE OF PROSTATECTOMY.*

By Edgar G. Ballenger, M. D., and Omar F. Elder, M. D.,
Atlanta, Ga.

So well established is the fact that prostatectomy affords greater relief than any other treatment for elderly men who suffer with urinary obstruction due to hypertrophy of the prostate, that no apology will be made for presenting this subject nor will your time be taken to give the facts that prove the advisability of this operation.

An important point to bear in mind constantly is the *age* and *infirmity* of the patients who need this operation; consequently the utmost care should be exercised in the preparation of the patient for this ordeal. The hazard would not be great if the operation were one upon healthy men in the prime of life. Usually, though, the patient's kidneys are more or less impaired by disease incident to back pressure or age. For this reason tests to determine and renal functional capacity are *very important* and treatment should be instituted to improve the kidneys and to stimulate their activity both before and after the operation. It is much

---

*Read before the Chattahoochee Valley Medical Association, Jan. 16-17, 1912.

easier to prevent suppression of urine than it is to restore the function of the kidneys after this has occurred.

Knowledge as to amount of urine passed in twenty-four hours, the amount of residual urine, the rapidity with which the quality increases when an additional amount of water is consumed, the amount of albumin, and tube casts, and especially the rapidity with which phenosulphonephthalein is excreted from the kidneys enable us to estimate their functional capacity sufficiently well to *anticipate* and thus *minimize* this greatest danger. For more than a year we have applied Rountree & Geraghty's functional test with phenosulphonephthalein and consider it the most reliable method yet devised. We have utilized this test in all operative conditions where there was doubt as to the kidneys and so far we have not been misled by it. The test described by the originators of the method is as follows:

"Twenty minutes to half an hour before administering the test, the patient is given 300 to 400 cc. of water in order to insure a free urinary secretion, otherwise delayed time of appearance may be due to lack of secretion.

"Under aseptic precautions a catheter is introduced into the bladder and the bladder completely emptied. Noting the time, 1 cc. of a carefully prepared solution of the phenolsulphonephthalein containing 6 mg. to the cc. is administered subcutaneously in the upper arm by means of an accurately graduated syringe. (We have used the Record 2cc. syringe, which is graduated in fifths of a cc.) It is our custom to draw the solution to the second division above the first cc. mark and then to expel the contents until the plunger is on a level with the division above the cc. mark and then inject accurately 1 cc. This avoids any possible error which might occur as a result of the presence of an air bubble.

"The urine is allowed to drain into a test tube in which has been placed a drop of 25 per cent. NaOH solution and the time of the appearance of the first faint pinkish tinge is noted.

"In patients without urinary obstruction the catheter is withdrawn at the time of the appearance of the drug in the urine, and the patient is instructed to void into a receptacle at the end of one hour and into a second receptacle at the end of the second hour.

"A rough estimate of the time of appearance can be made by having the patient void urine without the use of the catheter at frequent intervals. In prostate cases it is wise to have the catheter in place until the end of the observation. The catheter is corked at the time of the appearance of the drug in the urine and the cork is removed at the end of the first hour and at the end of the second hour, each time the bladder being thoroughly drained. On many of the patients of this type on whom our observations have been made, a retention catheter has been in use as part of the routine treatment on account of the residual urine.

"Each sample of urine is measured and the specific gravity taken. Sufficient NaOH (25 per cent.) is added to make the urine decidedly alkaline in order to elicit the maximum color. The color displayed in the acid urine is yellow or orange, and this immediately gives place to a brilliant purple-red color when the solution becomes alkaline. This soluton is now placed in a liter measuring flask and distilled water added to make accurately 1 liter. The solution is then thoroughly mixed and a small filtered portion taken to compare with the standard, which is used for all these estimations.

"The standard solution used for comparison consists of 3 mg. of phenolsulphonephthalein (or 1-2 cc. of the solution used for injection) diluted up to 1 liter and made alkaline by the addition of only one or two drops of 25 per cent. NaOH solution. This is a beautiful purplish red solution retaining its intensity of color for weeks, or for an indefinite period. The one solution, therefore, serves for an immense number of tests.

"One cup of the colorimeter (right) is half-filled with this standard solution used for comparison, which has just been described and the plunger lowered so that the indicator reads at 10. A varying quantity (depending on the intensity of the color) of the diluted urine is placed on the other cup and the plunger manipulated until the two haves of the field are of an identical intensity of color. The indicator of the left plunger is now read, the fraction, as indicated by the Vernier scale, being taken into account. The estimation of the quality present is then a question of simple arithmetic."

Satisfactory renal activity cannot as a rule be obtained without removal of the residual urine. This may be secured by the use of a retention catheter placed in the urethra or as the writers prefer, by the insertion of a trocar and cannula (after previous sterilization with idoine and cocainization at the suprapubic point), about 1-2 inch above the symphysis pubes. The cannula should be of sufficient caliber to admit a 12 F. catheter; if hemorrhage or a large amount of mucous is present a larger catheter should be used.

The bladder should be well distended and the patient requested to tighten the abdominal muscles while the trocar is being inserted. A cystoscopic examination may be made through the cannula to determine the presence of stones and the character of the prostatic enlargement. Such an examination causes less discomfort than does an ordinary cystoscopic examination and eliminates entirely the possibility of retention of the urine being made worse after the examination as a small catheter is inserted through the cannula, which is then removed and the catheter left in place for drainage of the bladder and irrigations (Belfield.) The catheter should be withdrawn until its distal end cannot pass into the deep urethra or a frequent desire to urinate will be produced. A suture passed through the skin at the site of the puncture and then through the catheter and tied, holds it in place. This simple and painless procedure is far preferable to a suprapubic incision, as ordinarily done in the two-way operation, for a number of good reasons: First, it can be done with less difficulty than the cystotomy and causes the patient less pain. Second, the amount of urine withdrawn can be accurately gauged and regulated so as not to drain off all at once if the distention is great. Third, the patient is not confined to his bed or his room by the operation and the clothing may be kept dry and clean. Fourth, ample drainage and means of medication are afforded by the catheter. Fifth, if the kidney function and the patient's health improve, prostatectomy may be done by either the suprapubic or the perineal route, as indicated. Sixth, if the patient's condition remains unsatisfactory this temporary procedure affords drainage and comfort, and by removing the catheter occasionally and replacing it with a new one, he may continue for months with this method of drainage. Of course if the amount of residual urine is small and the kidney function good, urethral or suprapubic drainage is unnecessary.

Affections of the lung should also be guarded against by selecting a time for the operation when the patient is free from colds and bronchitis; he should be allowed to wear his accustomed apparel when in bed, whether it be a red flannel undershirt and drawers or stiff bosomed white shirt. Changing from clothing the patient has ordinarily worn at night for years to a light cotton shirt that opens in the back and exposes his body much of the time undoubtedly favors pulmonary complications, which next to renal failure give us the greatest concern as to the patient's welfare. A short anaesthetic is of the utmost importance. Many times nitrous oxide gas and oxygen will afford satisfactory anaesthesia. It may not give sufficient muscular relaxation without a small amount of ether. Everything possible should be done before the anaesthetic is started so that no unnecessary delay is permitted on account of details which could easily have been arranged previously. A short anaesthetic and previous preparation of the kidneys are of more importance than the route used or the actual enucleation of the gland, provided the method selected is the one best suited to the pathologic conditions present and the enucleation is done between the sheath and the capsule. Next in importance comes the after treatment. Both the suprapubic and the perineal method have advantages and disadvantages. Generally speaking, the suprapubic is to be preferred when there are stones in the bladder, or when the enlargement is chiefly intravesical, or if the median portion is extensively hypertrophied, or the gland is a very large adenomatous one. The perineal is preferable for small fibrous prostates and for the larger ones which present toward the perineum and rectum rather than into the bladder. If there is no special reason for selecting either route, the patient will usually obtain a better result if the surgeon operates by his favorite method and the one he is most familiar with. We prefer the suprapubic method for various reasons we will not take the time to give.

In making the incision for the suprapubic operation, the important things to remember are to have the bladder well distended, to push up the fat and peritoneum with gauze so as not to enter the peritoneal cavity and to make the incision in the dome of the bladder rather than in the portion immediately posterior to the symphysis pubes, as healing progresses more satisfactorily when the bladder incision is so made (Squiers).

The finger-nail is used to score through the mucous membrane of the most prominent part of the prostate and the enucleating finger is kept carefully between the capsule proper of the gland and the enclosing sheath, enucleating from before backward. If the capsule is removed with the gland and the sheath is left behind the hemorrhage is greatly reduced, all of the gland is removed and the danger of entering the rectum and of infection is minimized. First one side then the other is enucleated, the lobes being removed together or singly. The finger is introduced into the deep urethra to determine if all of the obstruction has been removed. The bladder is flushed freely with hot physiologic salt solution and the cavity from which the prostate was removed is packed for a few moments with gauze saturated with 1 to 1,000 adrenalin chloride solution. After this is removed a two-way retention catheter (Dr. Sage Hardin) is placed in the urethra so that it drains the prostatic cavity. A good sized rubber tube is placed through the suprapubic incision, which is sewn together snugly, layer by layer. A penrose cover is placed in the space of Retzius for drainage. Plenty of sterile gauze and cotton are bound in place with the tube projecting about each inches for drainage into a bottle to be placed at the patient's side when he has been removed to his bed. he two-way catheter is allowed to drain into a sterile urinal placed between the patient's thighs.

Special nurses are secured and a few ounces of 1 to 10,000 adrenalin chloride in hot physiologic salt solution are injected through the catheter and the suprapubic tube every 15 minutes until the hemorrhage diminishes. In about 12 hours the time may be reduced to every 1-2 hour and the following day to one hour and finally to two or three times daily until the tube is removed. An ordinary bulb ear syringe, kept in a basin of bichloride, affords a convenient means of injecting the solution into the bladder. With the two-way catheter in the penis, the tube through the wound and the solution injected through them frequently there is little danger of their becoming blocked with clots.

In about a week the tube through the wound may be removed and irrigations continued daily through the opening and the catheter until the fistula heals.

If a careful examination shows that the operation may be done better by the perineal route, one of two methods of enucleation may be used. First an inverted "Y" or "V' incision may be made, the central tendon of the perineum exposed by blunt dissection, then incised, having a finger in the rectum to prevent injury to this viscus. With a sound now in place, an incision is made in the urethra at the apex of the prostate, a Young's retractor is inserted and opened. By blunt dissection and traction the prostate may be exposed and brought into view. An incision is now made over each lateral lobe, through which they are removed. Care is then taken to remove any median mass or other obstruction. After thorough irrigation with hot physiologic salt solution a large catheter is placed in the uethra for drainage and gauze drainage is left in each side from which the gland was removed. Second, if preferred for small glands a median perineal incision may be made and the urethra incised through its prostatic portion and each side enucleated without the exposing the gland by dissection. The incision is carefully brought together and dressed with abundant gauze and cotton. Frequent injections of salt solution are made through the catheter to wash out the bladder and free it from clots. After a few days the drainage gauze is removed from the wound.

The patients are always allowed to sit up at the earliest moment possible and are soon allowed in roller chairs on the porches if the weather permits.

No instrumentation is necessary after the operation, but copious bladder irrigations are advised until the wound heals and until the cystitis (if present) is cured. The seminal vesicles should not be disturbed if possible, by either operation. Sexual potentcy however does not depend upon the presence of the seminal vescicles, but rather upon a number of other factors such as age, previous condition, general health, etc. If lost before operation, it may or may not be restored; if present at the time of operation it may or may not return later.

Many facts of more or less importance have of necessity been omitted as a full discussion of the subject would have made the paper too long. The following points, however, demand emphasis: The functional capacity of the kidneys should be carefully estimated and suitable precautions taken to prevent suppression of urine and uremia after the operation. Every care should be taken to prevent pulmonary complications by measures that shorten the period of anaesthesia, and the use of nitrous oxide is advised when possible. The position of the patient when in bed should be frequently changed and he should be allowed up at an early date, depending upon the circumstances. The gland should be enucleated so that its capsule is removed with it and the sheath left behind. Ample drainage should be provided and frequent irrigations administered, especially until the hemorrhage has ceased.

---

### SURGICAL SUGGESTIONS.

In the presence of a tumor in the right iliac region it is rarely safe to exclude appendicitis from the diagnostic possibilities.

A chronic gonorrhea in women may be due to uncured disease of Skene's ducts. After these are slit open with a fine scapel or with the galvano-cautery knife, the disease will be cured.

In a case of abdominal disease a vague mass in the epigastrium associated with exaggerated distinctness of aortic pulsation at that point suggests pancreatic disease or retroperitoneal lymphatic tumor.

In carcinoma of the bowel local signs appear first and deterioration in health later; in intestinal sarcoma impairment of health is first noted and local signs appear later. In carcinoma obstructive symptoms are the rule, in sarcoma the exception. In carcinoma the growth of the tumor is relatively slow, in sarcoma it is rapid.

When vomiting and right iliac pain or tenderness are associated with a chill and a pyrexia of 105 degrees or 106 degrees, exclude pneumonia and malaria before operating for appendicitis. If the appendix is diseased, exclude mesenteric thrombosis before closing the wound.—American Journal of Surgery.

